# Association of IL-6 Polymorphism -174G/C and Metabolic Syndrome in Hypertensive Patients

**DOI:** 10.1155/2015/927589

**Published:** 2015-02-28

**Authors:** Andrei Alkmim Teixeira, Beata Marie Redublo Quinto, Maria Aparecida Dalboni, Cassio Jose de Oliveira Rodrigues, Marcelo Costa Batista

**Affiliations:** ^1^Nephrology Division, EPM, Universidade Federal de São Paulo (UNIFESP), 04039-032 São Paulo, SP, Brazil; ^2^Nove de Julho University, 01156-050 São Paulo, SP, Brazil; ^3^Nephrology Division, New England Medical Center, Tufts University, Boston, MA 02111, USA; ^4^Research and Education Institute, Hospital Israelita Albert Einstein, 05652-900 São Paulo, SP, Brazil

## Abstract

*Introduction*. Visceral obesity, the central core of metabolic syndrome (MetS), is conceived as the pathogenic basis of an increased cardiovascular burden and is related with changes in cytokines. We investigated whether IL-6-174G/C gene polymorphism is associated with MetS prevalence in hypertensive patients. *Method*. A population of hypertensive patients was included and stratified by the presence of MetS according to IDF criteria and evaluated by Framingham risk score. The IL-6-174G/C genotyping was performed by polymerase chain reaction and the prevalence of MetS was compared between “C” carrier and “non-C” carrier groups. *Results*. From an original sample of 664 patients, 612 (34.2% men, age 57.3 ± 10.1, 30.4% diabetics) were included. MetS was diagnosed in 51.3% of total population and “C” carriers demonstrated high prevalence of MetS (*P* < 0.05) and each of its components. On binary logistic regression, it was observed that the IL-6 polymorphism was independently associated with occurrence of MetS, even after adjusting for covariates (OR 1.13–2.37, 95% CI, *P* < 0.05). *Conclusion*. The C allele at the -174 locus of IL-6 gene is independently associated with the occurrence of metabolic syndrome, emphasizing the importance of inflammatory genetic background in the pathogenesis of visceral obesity and related cardiovascular burden.

## 1. Introduction

Metabolic syndrome (MetS), a disorder intimately associated with insulin resistance, increases the risk of cardiovascular diseases (CVD) or cerebrovascular events by three times and has an important impact on global mortality [1–10]. Visceral obesity, a core component of MetS, is considered the pathogenic basis of the burden of the syndrome and it is related to high levels of inflammatory cytokines [11]. In fact, the overproduction of cytokines is involved in the onset of an early inflammatory state and could be a significant prognostic indicator of risk of obesity and metabolic syndrome [12].

IL-6 is a mediator of inflammatory and immune responses, which also affects a variety of metabolic processes as an autocrine and paracrine regulator of adipocyte function [13, 14]. A single nucleotide polymorphism (SNP) common and specific to IL-6, -174G/C [15], has been related to altered levels of proinflammatory cytokines in humans, with C allele carriers exhibiting higher levels of IL-6. Although studies evaluating the relationship between cardiovascular disease and IL-6 polymorphism have produced contradictory results [16–21], most of them have pointed to the role of C alleles in inducing increased cardiovascular risk. In view of this evidence, we examined if the -174G/C polymorphism of the IL-6 gene is associated with the occurrence of MetS in hypertensive patients.

## 2. Materials and Methods

### 2.1. Population

Patients were selected from those treated at the Integrated Center for Hypertension and Cardiovascular Metabolism of the Federal University of São Paulo (UNIFESP). The most recent medical records were reviewed. Only participants classified as having hypertension were included. Patients were considered to have hypertension if they received antihypertensive drug treatment or if their blood pressure (BP) was 140/90 mmHg or higher. The study protocol was approved by the UNIFESP Ethics in Research Committee. All individuals participating in the study provided written informed consent. The study was conducted in accordance with the Declaration of Helsinki and with Brazilian National Ministry of Health Resolution CNS 196/96.

Patients were stratified by the presence of MetS according to International Diabetes Federation (IDF) criteria for MetS [22]. Lipid-lowering medications were discontinued in the four weeks preceding inclusion in the study as described: 3-hydroxy-3-methylglutaryl coenzyme A reductase inhibitors (statins); cholesterol absorption inhibitors such as ezetimibe, probucol, cholestyramine, niacin, and fibric acid derivatives (fibrates); and drugs for obesity treatment (orlistat and sibutramine). The Framingham risk score was used to evaluate cardiovascular risk [23]. Data regarding comorbidities, medication in use, and history of cardiovascular disease (CVD) were recorded. CVD was defined as the presence of coronary heart disease (evidence of silent myocardial infarction or myocardial ischemia, history of unstable angina or stable angina pectoris, and history of coronary angioplasty or coronary artery surgery) or coronary heart disease risk equivalents (peripheral arterial disease, abdominal aortic aneurysm, carotid artery disease, and renal artery disease), according to the American Heart Association guidelines [24]. All of these cardiovascular events were confirmed by medical records. Patients underwent anamnesis and physical examination, in which weight, height, BP, and waist circumference were determined. Body mass index (BMI) was calculated by dividing weight in kilograms by height in meters squared (kg/m^2^). BP was obtained by a trained operator with the patient in the sitting position after five minutes of rest. A mercury sphygmomanometer was used according to a standard protocol, and BP was calculated as the average after excluding the first of four measurements [25].

### 2.2. Laboratory Tests

Fasting glycemia and total serum cholesterol were determined using a colorimetric enzyme immunoassay. High-density lipoprotein cholesterol (HDL-C) levels were determined using a homogeneous colorimetric enzyme immunoassay, and low-density lipoprotein cholesterol (LDL-C) levels were calculated using Friedewald's formula. C-reactive protein (CPR) was determined using a chemiluminescence immunoassay (DPC Medlab, Los Angeles, USA), with analytical sensitivity of 0.01 mg/dL, intra-assay coefficient of variation of 4.2–6.4%, and interassay coefficient of variation of 4.8–10%. Serum creatinine levels were obtained using an alkaline picrate method that had been calibrated to be traceable to isotope dilution mass spectrometry (IDMS), as recommended by the National Kidney Disease Education Program [26]. Glomerular filtration rate (GFR) was estimated using the equation described in the Modification of Diet in Renal Disease (MDRD) Study, and only patients with values >30 mL/min were included in the evaluation. Nephelometry was used for apolipoprotein-A and apolipoprotein-B assays (HITACHI 9021 analyzer, ROCHE, USA).

### 2.3. IL-6-174G/C Genotyping by PCR-SSP Using the SSP DNA Typing Kit

Genomic DNA was prepared from peripheral blood using standard techniques. The -174G/C SNP was detected by polymerase chain reaction with sequence-specific primers (PCR-SSP) using the Cytokine Genotyping Primers Kit (One Lambda, Canoga Park, USA) according to the manufacturer's instructions. In short, a 175-bp fragment of the IL-6 gene was amplified. Gel electrophoresis on a 2% agarose gel revealed either a positive or negative specific amplification for each well. Subsequently, the results were entered in the cytokine worksheet provided with the kit manual. Quality checks to ensure correctness of the genotypes were carried out by independent rating of the results by two or three investigators. Discrepancies were resolved by either reaching a consensus or regenotyping.

### 2.4. Statistical Analysis

Student's *t*-test using 2-tailed analysis for independent means was used for intergroup comparisons of quantitative variables, and Mann-Whitney test was used for qualitative variables. Chi-squared test was used to compare proportions. The results are presented as means and standard deviations. Allele frequencies were estimated by gene counting. A binary logistic regression model was used to identify the association of the presence of the IL-6 single nucleotide polymorphism -174G/C with occurrence of MetS, after adjusting for the effects of sex, age, race, and LDL-C and Framingham score. Statistical analysis was carried out using the SPSS version 22.0 software.

## 3. Results

Starting with a cohort of 664 patients, 52 were excluded due to incomplete genotyping or not fulfilling the diagnosis of hypertension. A total of 612 patients (34.2% men, age 57.3 ± 10.1, 30.4% diabetics) were included in the analysis. Overall, 10.1% had a history of CVD and 27.4% showed a family history of CVD.

**Figure 1:**
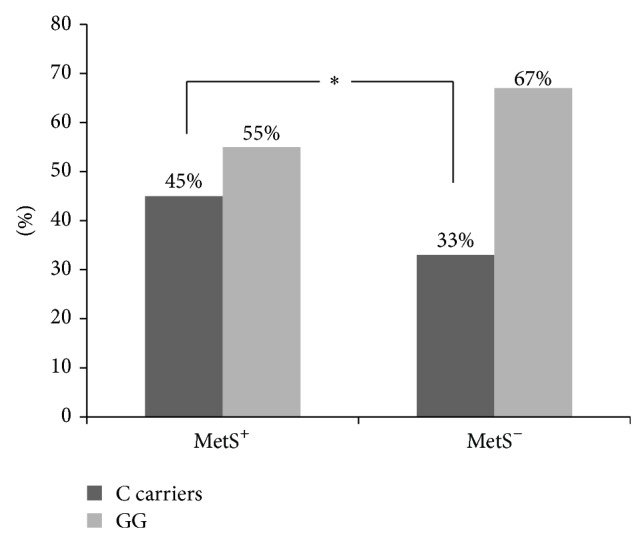
MetS^+^ and MetS^−^ IL-6 genotype frequency. ^*^(*P* < 0.05).

Table 1 shows the baseline characteristics of the total population and of the 314 (51.3%) patients with and 298 (48.7%) without metabolic syndrome (MetS^+^ and MetS^−^), respectively. IL-6 genotype prevalence is demonstrated in Table 2. The G/G carriers were more prevalent in all groups, when compared to the GC and C/C genotypes. When grouped together into two distinct IL-6 genotype groups, C carriers (including G/C and C/C genotypes) and GG genotype, the C carriers group accounted for 39.1% of the population studied. As shown in Figure 1, there was increased prevalence of MetS in the C carriers group when compared with the GG group.

Considering each component of MetS, there were significant differences when comparing the groups. As shown in Table 3, the C carrier group demonstrated higher levels of BMI, waist circumference, and VLDL-C and lower levels of HDL-C and Apo-A. Higher levels of glomerular filtration rate and total leukocytes were also found in the group of C allele carriers.

In binary regression analysis performed to assess the independent relationship between IL-6 polymorphism and MetS, the presence of the C allele, even corrected for confounders and other clinical variables, was a significant and independent predictor of MetS, as shown in Table 4.

## 4. Discussion

The incidence of MetS is rapidly approaching epidemic levels. Population studies have shown that this syndrome plays a pivotal role in the occurrence of CVD [27, 28]. In a cohort of patients with hypertension, we demonstrated that “C” carriers have higher prevalence of metabolic syndrome than noncarriers. Mostly, “C” carriers showed higher prevalence of each of the components of MetS. In fact, BMI, waist circumference, CRP, and VLDL-C values were higher and HDL-C and Apo-A levels were lower in this group. Some clinical studies have analyzed the association between the IL-6 polymorphism and cardiovascular risk factors and metabolic changes. We observed high prevalence of MetS, and as expected, BMI, systolic and diastolic blood pressure, waist circumference, and CRP values were higher and HDL-C and APOA were lower in the MetS^+^ versus MetS^−^ group. These findings go along with the concept that MetS is associated with low-grade inflammation [29], since interleukin-6 (IL-6) is a key proinflammatory and immune-stimulatory cytokine of presumed importance for CVD. The mechanisms underlying the pathogenesis of obesity-related atherosclerosis are yet to be clarified.

The genotype distribution of the IL-6 polymorphism has been described in native Americans, Spanish Caucasian patients [30], and 3052 UK healthy men as well [31], with higher G allele frequency in all these populations. The G/G genotype was more prevalent in diabetic patients. Accordingly, in our study, we demonstrated marked prevalence of the G/G genotype compared to G/C and C/C, even in the MetS^+^ and MetS^−^ groups. When grouped together into two distinct IL-6 genotype groups, C carriers and G/G genotype, the C carrier genotypes comprised 45% of the MetS population.

Berg et al. associated C carriers with higher coronary artery disease risk [16], while Revilla et al. associated them with stroke [17], with higher prevalence of cardiovascular risk factors such as hypertension in Losito et al. [18] and type 2 diabetes mellitus (T2DM) in Kristiansen and Mandrup-Poulsen [19]. Nevertheless, it seems that in obese children the C allele carriers have less subcutaneous fat and higher HDL-C concentrations [20], in contrast to the association of the IL-6 polymorphism -174G/C and obesity. Still, the IL-6 promoter polymorphism -174G/C has been related to the development of obesity and MetS. With regard to the anthropometric aspect, C allele-carrying patients seemed to have higher Lp (a) and CRP levels as well as increased BMI. Moleres et al. studied 504 young adults and found that this polymorphism influences the association of adiposity and some plasma markers [21]. Similarly, we demonstrated that both BMI and waist measures were higher in the C carrier group than in the G/G group, as well as higher CRP levels in the C carrying patients. However, in a specific kidney transplant population with overweight [32], IL-6 concentrations were lower in the C carrier group, and the incidence as well as the risk of the development of atherosclerotic events was higher in the G/G group. Similarly, other studies have described altered levels of circulating IL-6 related to the IL-6 polymorphism, with contradictory results. Besides, the* Nurses' Health Study and the Health Professional Follow-up Study*, including 2691 diabetics and 3237 control subjects, demonstrated that there is no data to support a substantial association between IL-6 polymorphisms and circulating IL-6 levels [33]. In view of the lack of evidence of a relationship between circulating IL-6 levels and cardiovascular risk factors, we decided not to evaluate these data in our study.

Therefore, chronic inflammation could represent a triggering factor in the origin of MetS, with cytokine hypersecretion, leading to insulin resistance and diabetes in genetically or metabolically predisposed individuals. Alternatively, resistance to the anti-inflammatory actions of insulin would result in enhanced circulating levels of proinflammatory cytokines resulting in persistent low-grade inflammation [34]. It seems that white adipose tissue produces and releases cytokines and other factors that could be responsible for the chronic inflammatory state of obesity [35]. De Lorenzo et al. [12] demonstrated that proinflammatory cytokine concentrations were elevated in normal weight women and preobese women and that the circulating concentrations were correlated with percent fat mass. This overproduction of cytokines would be involved in the onset of the early inflammatory state and could be a significant prognostic indicator of risk of obesity and MetS. Along the same line, di Renzo et al. [36] also demonstrated that this polymorphism is a marker that could help to identify vulnerable individuals at risk of age- and obesity-related diseases.

Similarly, some authors have identified an association of C allele presence and cardiovascular risk factors, like obesity [37, 38] and hyperinsulinemia/insulin resistance, represented by HOMA index [39, 40]. Consequently, C allele has been associated with major cardiovascular risk in some other publications. This way, we have described that, in hypertensive patients, this polymorphism might influence the prevalence of Mets criteria and, consecutively, Mets prevalence.

This study had some limitations. The study population consisted of hypertensive patients with at least one metabolic syndrome criterion. Therefore, our results cannot be generalized, as the study was restricted to treated hypertensive patients. However, since hypertension is a disease with high prevalence in the general population, we believe that it is particularly important to assess cardiovascular risk in these patients. For ethical reasons, antihypertensive drugs were not interrupted, thereby compromising the study of the relation between blood pressure and IL-6 polymorphism. This fact may have precluded the demonstration of blood pressure differences between groups. In contrast, all patients were not on lipid-lowering treatment, allowing lipid profile comparisons. This was a cross-sectional study, without the possibility to properly infer causality. Nevertheless, since our results demonstrated high prevalence of MetS in C carrier patients, these findings suggest that IL-6 polymorphism may be a risk factor for Mets.

## Figures and Tables

**Table 1:** Clinical and biochemical characteristics of MetS^+^ and MetS^−^ populations.

	Total Population	MetS^+^	MetS^−^	*P* ^*^
		314	298	
Age (years)	57.3 ± 10.1	57.8 ± 9.4	56.6 ± 10	NS
Gender (% men)	34.2	38.1	30.7	<0.05
BMI	29.4 ± 5.2	31.4 ± 4.9	27.2 ± 4.7	<0.01
SBP (mmhg)	136.2 ± 19.7	138.3 ± 18.5	133.9 ± 20.6	<0.01
DBP (mmhg)	84.7 ± 11.5	85.7 ± 11.2	83.7 ± 11.1	<0.05
Waist (cm)	95.9 ± 12.2	101.1 ± 10.5	90.3 ± 11.5	<0.01
Glu (mg/dL)	103.3 ± 42.9	115.1 ± 45.6	90.3 ± 35.4	<0.05
Chol (mg/dL)	211.2 ± 41.2	213.1 ± 44.2	209.1 ± 37.6	NS
LDL-C (mg/dL)	123.6 ± 35.8	124.5 ± 37.0	122.7 ± 34.4	NS
HDL-C (mg/dL)	57.4 ± 15.6	52.3 ± 13.1	63.0 ± 16.2	<0.01
VLDL-C (mg/dL)	28.3 ± 13.1	33.7 ± 13.2	22.7 ± 10.6	<0.01
TG (mg/dL)	152.9 ± 95.0	186.6 ± 107.4	115.6 ± 60.0	<0.05
GFR (mL/min)	79.2 ± 24.9	83.6 ± 25.9	74.4 ± 28.8	<0.01
Total leukocytes (mm^3^)	6250 ± 1887	6520 ± 1828	5953 ± 1910	<0.01
CRP (mg/dL)	0.57 ± 0.69	0.68 ± 0.73	0.44 ± 0.62	<0.01
APO-A (mg/dL)	141.9 ± 25.4	136.1 ± 23.3	146.1 ± 26.2	<0.01

BMI: Body mass index; SBP: Systolic blood pressure; DBP: Diastolic blood pressure; Glu: glucose; Chol: Total cholesterol; LDL-C: Low density lipoprotein cholesterol; HDL-C: High density lipoprotein cholesterol; VLDL-C: Very low density lipoprotein cholesterol; TG: Triglycerides; GFR: Glomerular filtration rate; CRP: C-reactive protein; APO-A: Apolipoprotein A. ^*^MetS^+^ versus MetS^−^.

**Table 2:** IL-6 genotype frequency.

	GG	GC	CC
Total	373 (60.9%)	181 (29.6%)	58 (9.5%)
MetS^+^	174 (55.4%)	105 (33.5%)	35 (11.2%)
MetS^−^	199 (66.8%)	76 (25.5%)	23 (7.7%)

MetS^+^: Metabolic syndrome patients, MetS^−^: Non-metabolic syndrome patients.

**Table 3:** Characteristics of different IL-6 genotype populations according to the presence of C carriers.

	C carriers	GG	*P*
	*N* = 239	*N* = 373	
Age (years)	56.9 ± 10.4	57.1 ± 10.1	NS
Gender (% men)	20	13.2	NS
BMI	30.1 ± 5.4	28.9 ± 5.1	<0.05
SBP (mmhg)	138 ± 20.6	135 ± 19.5	NS
DBP (mmhg)	85.8 ± 11.3	84.2 ± 11.3	NS
Waist (cm)	97.1 ± 12.2	95.0 ± 12.3	<0.05
Glu (mg/dL)	105.1 ± 42.1	101.9 ± 44.3	NS
Chol (mg/dL)	211.7 ± 37.2	211.9 ± 43.4	NS
LDL-C (mg/dL)	124.2 ± 32.0	123.8 ± 37.9	NS
HDL-C (mg/dL)	55.4 ± 15.3	58.7 ± 15.4	<0.05
VLDL-C (mg/dL)	30.5 ± 13.4	27.1 ± 13.1	<0.05
TG (mg/dL)	162.6 ± 91.8	149.0 ± 100.6	NS
GFR (mL/min)	82.6 ± 26.9	77.7 ± 23.7	<0.05
Total leukocytes (mmm^3^)	6636 ± 1943	5977 ± 1770	<0.05
CRP (mg/dL)	0.67 ± 0.73	0.50 ± 0.63	<0.05
APO-A (mg/dL)	137.6 ± 25.4	143.3 ± 25.4	<0.05
APO-B (mg/dL)	107.4 ± 21.6	104.2 ± 26.0	NS
CVD	8.8 ± 28.3	10.2 ± 30.2	NS

BMI: Body mass index; SBP: Systolic blood pressure; DBP: Diastolic blood pressure; Glu: glucose; Chol: Total cholesterol; LDL-C: Low density lipoprotein cholesterol; HDL-C: High density lipoprotein cholesterol; VLDL-C: Very low density lipoprotein cholesterol; TG: Triglycerides; GFR: Glomerular filtration rate; CRP: C-reactive protein; APO-A: apolipoprotein A; APO-B: apolipoprotein B; CVD: Cardiovascular disease.

**Table 4:** Association of MetS^+^ and IL-6 C carriers.

	OR	95% CI	*P*
Metabolic syndrome			
Non adjusted	1.6	1.15–2.23	0.006
Adjusted for sex, age	1.62	1.16–2.26	0.005
Adjusted for sex, age, race	1.47	1.04–2.09	0.031
Adjusted for sex, age, race and CVD	1.52	1.07–2.17	0.021
Adjusted for sex, age, race, CVD and LDL-C	1.68	1.17–2.40	0.005
Adjusted for sex, age, race, CVD and LDL-C and Framingham score	1.64	1.13–2.37	0.009

CVD: cardiovascular disease, LDL-C: Low density lipoprotein cholesterol.

## Abbreviations

Mets: Metabolic syndrome
CVD: Cardiovascular disease
IL-6: Interleukin-6
SNP: Single nucleotide polymorphism
UNIFESP: Federal University of São Paulo
BP: Blood pressure
IDF: International Diabetes Federation
BMI: Body mass index
HDL-C: High-density lipoprotein cholesterol
LDL-C: Low-density lipoprotein cholesterol
VLDL-C: Very low-density lipoprotein cholesterol
PCR: Polymerase chain reaction
GFR: Glomerular filtration rate
MDRD: Modification of Diet in Renal Disease
PCR-SSP: Polymerase chain reaction with sequence-specific primers
G/G: Guanidine-guanidine
G/C: Guanidine-cysteine
C/C: Cysteine-cysteine
Apo-A: Apolipoprotein-A
UK: United Kingdom
T2DM: Type 2 diabetes mellitus
Lp (a): Lipoprotein-a.
